# Preparation Methods for Improving PEEK's Bioactivity for Orthopedic and Dental Application: A Review

**DOI:** 10.1155/2016/8202653

**Published:** 2016-04-04

**Authors:** Davood Almasi, Nida Iqbal, Maliheh Sadeghi, Izman Sudin, Mohammed Rafiq Abdul Kadir, Tunku Kamarul

**Affiliations:** ^1^Department of Materials, Manufacturing and Industrial Engineering, Faculty of Mechanical Engineering, Universiti Teknologi Malaysia, 81310 Skudai, Johor, Malaysia; ^2^Medical Implant Technology Group (MEDITEG), Faculty of Bioscience and Medical Engineering, Universiti Teknologi Malaysia, 81310 Skudai, Johor, Malaysia; ^3^Faculty of Chemical and Energy Engineering, Universiti Teknologi Malaysia, 81310 Skudai, Johor, Malaysia; ^4^Department of Orthopaedic Surgery, NOCERAL, Faculty of Medicine, University of Malaya, 50603 Kuala Lumpur, Malaysia

## Abstract

There is an increased interest in the use of polyether ether ketone (PEEK) for orthopedic and dental implant applications due to its elastic modulus close to that of bone, biocompatibility, and its radiolucent properties. However, PEEK is still categorized as bioinert due to its low integration with surrounding tissues. Many studies have reported on methods to increase the bioactivity of PEEK, but there is still one-preparation method for preparing bioactive PEEK implant where the produced implant with desirable mechanical and bioactivity properties is required. The aim of this review is to present the progress of the preparation methods for improvement of the bioactivity of PEEK and to discuss the strengths and weaknesses of the existing methods.

## 1. Introduction

PEEK with high chemical resistance, radiolucency, mechanical characteristics compared to those of human bones [1–6], and local inflammation and stress shielding problem of the metallic implant [7, 8] has become a very interesting biomaterial for scientists and a promising good alternative for metallic implants. The radiolucency of PEEK is vital especially for the postoperative radiotherapy follows the surgical removal of the tumor. The presence of metallic implants can change the local dose distribution [9, 10]. In addition, it can be repeatedly sterilized and shaped by machining and heat contouring to fit the contour of bones [11]. PEEK has been used for load bearing orthopedic applications such as spinal cage, dental implant, and screws [12, 13]. Despite these excellent properties, PEEK is still categorized as bioinert due to its very low reaction with the surrounding tissue, which limits its potential applications [1]. For overcoming this problem, several methods have been proposed which can broadly be divided into two main categories: incorporation of bioactive materials such as hydroxyapatite (HA) and titanium dioxide (TiO_2_) into PEEK composite and surface treatment techniques such as laser surface modification, coating with the bioactive material, and wet chemical treatment [14–22].

A review of presently available methods to improve the bioactivity of PEEK was conducted with the aim of providing sufficient information regarding known preparation techniques and to compare the pros and cons of each of these methods. It is hoped that this will lead to a better understanding of the methods available and a clear reason as to why a method should be ultimately chosen by a researcher or an implant manufacturer.

## 2. PEEK's Bioactivity

One of the important factors that lead to successful implantation is the biological response to the implant, which very much depends on the bioactivity of the implant. A material is considered bioactive if it obtains a particular biological answer to the interface of the element, which ends in the formation of a bond between the tissue and the substance [23].

When an implant is placed in the body, molecules of water are one of the first molecules to reach the implant surface. The absorption of proteins on the surface is influenced by the initially adsorbed water molecules and is affected by surface structure, chemistry, charge, and wettability [24]. Subsequently, these adsorbed proteins influence cellular interactions and eventually tissue growth [25, 26]. Surfaces with moderate hydrophilicity properties showed the best interactions with cells and surrounding tissues [27].

**Figure 1:**
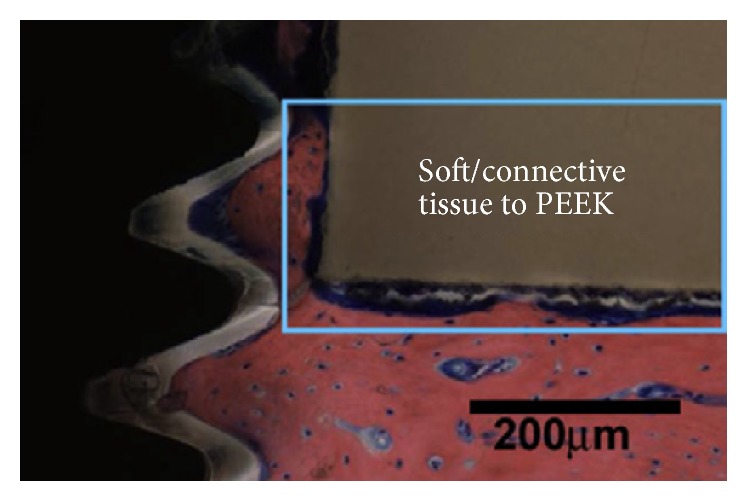
PEEK bioinert properties and growth of soft tissue around it [30].

An essential problem with most polymers, including PEEK, is their low-surface energy. This hydrophobic property of the surface can reduce cellular adhesion. The lack of response from the biological environment caused PEEK to be categorized as bioinert [28, 29]. As explained above one of the most important applications of PEEK is for orthopedic area. The bioinert properties of PEEK in the orthopedic area mean the growth of soft tissues around the PEEK implant instead of bone growth (Figure 1) [30].

**Figure 2:**
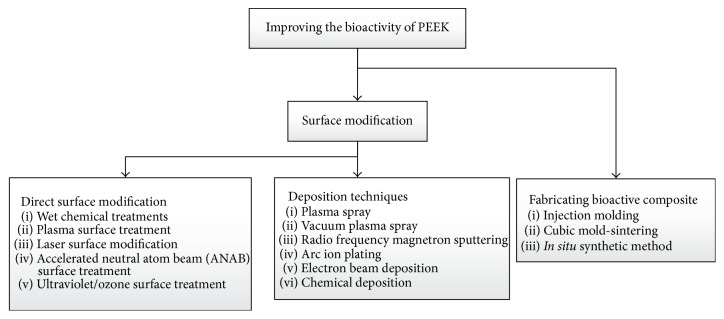
General categorization of the techniques for improving PEEK's bioactivity.

By changing the surface energy of the polymer, the reactions of the surrounded tissue to the polymeric implant can be improved, which could broaden its applications in the medical field, where direct bone interaction is important. Many methods have been used to alter the surface energy, and these methods can be broadly divided into two groups: compounding PEEK with a bioactive material and producing a composite and through surface modification.  Figure 2 shows the general categorization of the existing methods for improving the bioactivity of PEEK.

## 3. Surface Modification of PEEK's Implant

Surface modification is a series of approaches which alter the properties of the surface of the material but do not affect the bulk properties of the material. Surface modification methods can be broadly divided into two broad categories: direct surface modification and deposition methods.

### 3.1. Direct Surface Modification

Direct surface modification methods are techniques that changed the surface properties of the material without depositing any layer of new material on the surface. These techniques consist of the following.

#### 3.1.1. Wet Chemical Treatments

This is a method which is based on changing the surface chemistry of the implant and affects the bioactivity of the surface. Several studies reported that the bioactivity of PEEK could be increased by wet chemical treatment. Various chemical treatments modifying PEEK surface chemistry to PEEK-ONa, PEEK-OH, PEEK-F, and PEEK-OH (CFCl_3_) showed a decrease in water contact angle of the implant and, therefore, increase the bioactivity of PEEK [31]. Another study showed that the amine and carboxyl functional group on the surface of PEEK could improve cellular adhesion and growth [28].

*In vitro* study on Fibronectin (FN) adsorption for probing the bioactivity of PEEK-OH, PEEK-NH_2_, and PEEK-NCO produced by wet chemical treatment showed protein can merely be adsorbed onto PEEK-NCO that Fibronectin covalently grafted to PEEK-NCO [32]. The performances of the FN-grafted substrate improved adhesion and spreading of Caco-2 cells in the absence of serum in comparison with PEEK substrates, which were simply coated with FN [33]. In another study, wet chemical treatment was used as a pretreatment for enhancement of apatite formation via immersion in SBF. The effect of NaOH pretreatments on apatite formation of PEEK in SBF showed the growth of apatite coating layer was enhanced with NaOH pretreatment [34].

A recent study probed the effect of sulphonation and the production of 3D porous and nanostructured network on* in vitro* cellular behavior and* in vivo* osseointegration and apatite formation. Two types of sulphonated PEEK (SPEEK) samples, SPEEK-W (sulphonated PEEK with just subsequent water immersion) and SPEEK-WA (SPEEK-W with additional acetone immersion) were probed. They showed new bone can grow and penetrate the porous sulphonated layer. The SPEEK-WA samples showed better cytocompatibility, bioactivity, osseointegration, and bone-implant bonding strength [22].  Table 1 presents the summary of the existing functional groups which have been deposited on PEEK via wet chemical deposition to enhance the bioactivity of it.

#### 3.1.2. Plasma Surface Treatment

Plasma is often known as the fourth state of matter in which the gases are ionized and electrons are separated from their atoms. There are two types of plasma, hot plasma and cold plasma. In hot plasma using very high temperature, the gas is ionized. In cold plasma, the gas is ionized using low pressure in ambient temperature. The plasma can be used for altering the surface chemistry of the material. Plasma treatment of PEEK in oxygen, air, nitrogen, ammonia, and argon showed increasing of the wettability [35, 36].

*In vitro* study with osteoblast cells and wettability study carried out on plasma treated PEEK in N_2_/O_2_ showed the plasma treatment of PEEK reduced the water contact angle.* In vitro* study with osteoblast cells showed the plasma treatment does not have disadvantages on cell viability [37]. Plasma treated PEEK in NH_3_ showed lower water contact angle and increased cell growth [38]. Osteoblast biocompatibility test showed required biocompatibility for plasma treated PEEK in ammonia/argon and hydrogen/argon. Higher rate of cell proliferation and lower contact angle were demonstrated for plasma treated PEEK in comparison with untreated PEEK [39]. Plasma treatment of PEEK in chamber of CH_4_/O_2_ gas mixture showed better cell adhesion and lower water contact angle [40].* In vivo* study of oxygen plasma, modified PEEK in cortical and cancellous bone of the sheep showed an increase in push-out force test and the percentage of the bone-implant contact area in comparison of untreated PEEK [41].* In vitro* study via osteoblast precursor cells MC3T3-E1 and rat bone mesenchymal stem cells on plasma immersion ion implantation treatment with a gas mixture of water vapor as a plasma resource and argon as an ionization assistant of PEEK showed improvement of osteoblast adhesion, spreading, proliferation, and early osteogenic differentiation [42]. Also tuned PEEK by argon plasma treatment showed increasing of the surface roughness in comparison with pristine PEEK. As a consequence due to higher surface roughness and changing the surface chemistry of the treated PEEK, significant enhancements in terms of cell adhesion, proliferation, and metabolic activity were observed when compared to pristine PEEK [43]. Probing the effect of plasma treatment of PEEK by O_2_/Ar or NH_4_ on adhesion, proliferation, and osteogenic differentiation of adipose tissue-derived mesenchymal stem cells (adMSC) showed an improvement of bioactivity of plasma treated samples in comparison with nontreated samples [44].  Table 2 summarizes the ionization assistants which have been used for enhancement of the bioactivity of PEEK via plasma surface treatment method.

#### 3.1.3. Laser Surface Modification

Laser is a high energy photon source which can alter the surface roughness and wettability of the polymers. Laser treatments are used due to their low cost, high resolution, high-operating speed, and the fact that lasers do not change the bulk properties of implant. For these reasons, lasers become very interesting for scientists in order to improve the surface energy of the implants [45, 46]. This surface treatment technique can modify the surface chemistry of PEEK [47, 48]. Investigation into the effect of laser wavelengths on the wettability of PEEK showed the capability of this method in increasing the wettability of the PEEK for biomedical applications [49].

#### 3.1.4. Accelerated Neutral Atom Beam (ANAB) Surface Treatment

This technique is a method which is used to enhance the bioactivity of PEEK and improve the bone-implant integrity. In this technique a powerful beam of cluster-like packets of accelerated unbonded neutral argon (Ar) gas atoms is used to modify the surface of PEEK. The results showed that ANAB treatment of PEEK modified the surface in the nanometer scale, increased surface wettability, and improved human osteoblast cell proliferation to a level comparable with titanium. The* in vivo* study shows the bone tissue formation on the ANAB treated PEEK while no growth of bone tissue on the untreated PEEK was observed [50]. The atomic force microscope examination showed the effect of ANAB technique in producing nanoscale texturing on the surface.* In vitro* study of ANAB treated PEEK showed better osteoblast cell adhesion in comparison with untreated PEEK [51].

#### 3.1.5. Ultraviolet/Ozone Surface Treatment

Polymers can be degraded by exposure to sunlight because of the chemical reaction activation due to short wavelengths of ultraviolet (UV) of sunlight and photon-activation cross-linking or fragmentation of the polymer. UV/ozone treatment method for PEEK was used to change the surface energy of PEEK. The results showed increasing of the surface wettability of the treated PEEK by UV/ozone [52].

### 3.2. Deposition Techniques

Several methods exist for depositing bioactive material on PEEK such as plasma spraying, vacuum deposition, sol gel, dip coating, and immersion in SBF method [53]. In this section, the trend of progress of PEEK's coating is described based on the coated materials.

Hydroxyapatite is one of the most important materials which have been used widely for coating of biomaterials. HA coating on carbon fiber reinforced PEEK (CF/PEEK) via plasma spray method showed low adhesion of the coating layer to the substrate [54]. The authors explained that the high temperature used in plasma spray method caused the evaporation of the PEEK substrate preventing close contact between coating layer and substrate. In the next study, they coated titanium intermediate layer via vacuum-plasma-sprayed and after that coated hydroxyapatite layer on CF/PEEK for increasing the adhesion between the coating layer and the substrate. The cross section study showed very good interlocking between the PEEK substrate and the intermediate Ti layer [55]. To prevent damage to the PEEK substrate due to the high temperature during the coating process and damage to the PEEK substrate during the sintering, intermediate coating layer of yttria-stabilized zirconia (YSZ) was first deposited onto PEEK and after that the HA coating layer was deposited via radio frequency magnetron sputtering method. For increasing the adhesion between the substrate and coating layer, preplasma treatment was used for substrate. Microwave was used for sintering and forming crystalline HA coating layer. The authors showed the crystalline YSZ layer encouraged the HA layer during the sintering procedure by providing nucleation site for HA grain formation [56]. Hydroxyapatite coating via plasma spraying method on different PEEK (unfilled and carbon fiber reinforced composite) specimens was studied and chemical, crystallographic compositions, adhesions, and microstructures of HA coating via plasma spraying method on different PEEK (unfilled and CF/PEEK) specimens and comparison with HA coating on Ti-6Al-4V showed almost the same structure of HA coatings for PEEK and Ti-6Al-4V substrate. Mechanical tests showed the plasma spraying method does not have a negative effect on mechanical properties of PEEK implant [57].* In vitro* study with human bone marrow mesenchymal stem cells of HA coated PEEK via cold spray method showed early cell adhesion, viability improvement, and increased cell differentiation and proliferation.* In vivo* study on rabbits showed promotion of bone growth and integrity with the implant after coating [58]. HA coating on medical-grade PEEK via aerosol deposition showed dense microstructure with no pores and cracks with high-adhesion strength of HA coating layer without damaging the PEEK substrate.* In vitro* and* in vivo* study in terms of cell proliferation, differentiation, adhesion morphology, and bone-implant contact ratio showed enhancement for HA coated sample in comparison to uncoated PEEK [59].* In vivo* osseointegration (histomorphometry) study of surface modified PEEK implants showed the nano-HA coated implants have more bone area and more bone-to-implant contact in comparison to uncoated PEEK [60]. In our recent study the HA crystalline particles were chemically deposited on the PEEK's surface whereby crystallization process and high temperature for deposition of the HA were eliminated. For depositing the HA particles, the surface of the PEEK was sulphonated first to establish the –SO_3_H functional group, and then the polarity property of the HA particles was used to attach the particles to the functional group. The surface treatment was able to decrease the water contact angle from 72 to 36.4 degrees [61].* In vitro* study comprising apatite formation via SBF immersion and mesenchymal stem cell proliferation confirmed enhancement of bioactivity of treating PEEK via this method [62].

*In vitro* osteoblast study of PEEK substrate coated with TiO_2_ via arc ion plating method showed a significant improvement in cell adhesion, proliferation, and differentiation compared with an uncoated PEEK substrate [21]. The anatase-rich titanium dioxide (A-TiO_2_) and especially rutile-rich titanium dioxide (R-TiO_2_) intermediate layer onto the PEEK substrate showed enhancement of produced HA layer after immersion in SBF in comparison with pure PEEK. The authors explained that the intermediate layer, by providing nucleation site for growing HA, improves the produced HA layer. Osteocompatibility evaluation showed the produced HA layer improves osteocompatibility, in which R-TiO_2_ achieves the best result [63]. In another study the bone morphogenetic protein-2 (BMP-2) was immobilized on porous TiO_2_ coating layer on PEEK. The bone-to-implant contact ratio study showed better interaction of TiO_2_/BMP-2 coating layer in comparison with TiO_2_, and BMP-2 coating layer and pure PEEK [64].

*In vivo* study on sheep was performed on titanium plasma spray coating on the PEEK screw. Histological investigation showed higher bone-to-implant contact and lower soft tissue around coated samples in comparison with pure PEEK [65]. Electron beam deposition of Ti on PEEK produced a dense coating layer at low temperature.* In vitro* study in terms of proliferation and differentiation of MC3T3-E1 cells showed more than double improvement after Ti coating in comparison with pure PEEK.* In vivo* study showed that the bone-to-implant contact ratio increased with coating Ti on the PEEK substrate [6]. In another study the vacuum-plasma-sprayed Ti coating layer on CF/PEEK substrate was treated by sodium hydroxide (NaOH) solution for improving its bioactivity.* In vitro* study via SBF showed apatite formation on the coated samples while no apatite was formed on the untreated PEEK samples [66].* In vivo* comparative study for probing the effect of two different methods of PVD and VPS for deposition of the Ti on CF/PEEK screws showed no significant difference between these two methods in terms of bioactivity. The coated screws by these two methods showed better bone deposition and higher removal torque in comparison with uncoated screws [67].

An* in vivo* study of Ti-coated CF/PEEK for dental implant application via plasma vapor deposition was carried out to evaluate the bioactivity of Ti-coated CF/PEEK. The results showed direct growth of new bone for both coated and uncoated PEEK samples, but the coated samples showed better bone growth around the coated implant. However, the push-out test revealed almost the same interface strength between the coated and uncoated samples by new bone growth [68]. In another study, electron beam deposition method was used to deposit pure titanium on PEEK. The Ti coating layer showed superb adhesion properties to the PEEK substrate. Contact angle analysis showed the Ti coating enhances the wettability of PEEK.* In vitro* study by MC3T3-E1 cells for methoxyphenyl tetrazolium salt (MTS) assay to measure the proliferation of the cells shows enhancement of more than double for coated samples. Alkaline phosphatase (ALP) assay showed double differentiation level of cells for Ti-coated samples. Furthermore, an* in vivo* animal study showed much higher bone-in-contact (BIC) ratio for Ti-coated PEEK samples in comparison with the pure PEEK samples [6].

Zirconium and titanium tetra(tert-butoxides) are another bioactive material which was deposited on the surface PEEK at room temperature via vapor deposition to enhance the bioactivity of PEEK. The deposited metal layer reacted with the phosphonic acid for attachment of monolayer phosphonates.* In vitro* study showed significant enhancement of osteoblast cell growth as compared to the untreated surface [69]. Diamond-like carbon (DLC) is another material which was used to coat PEEK implant for increasing bioactivity.* In vitro* study via osteoblast showed better attachment, proliferation, and differentiation on DLC-coated PEEK compared to uncoated PEEK [70].  Table 3 presents the summary of the existing deposition methods/materials which have been used for enhancement of PEEK bioactivity.

## 4. Bioactive PEEK Composites

As explained before compounding with bioactive material is one strategy to increase the bioactivity of the PEEK implants. Different bioactive material such as HA, strontium-containing hydroxyapatite, TiO_2_, *β*TCP, and bioactive glass was compounded with PEEK for increasing the bioactivity of PEEK's implant. PEEK composites were produced for different applications. The most important application is load bearing implant application [71], but several other studies were carried out to show the feasibility of producing three-dimensional porous scaffold PEEK/HA for tissue engineering application [72–74] and cervical spinal fusion cages [75]. One of the most significant disadvantages of the PEEK composites is the low mechanical properties in comparison with PEEK [76–78]. Thus previous studies focused on probing the effect of different parameters on two important aspects of mechanical properties and bioactivity. In this part, previous studies of the PEEK composites were first broadly categorized as bioactivity and mechanical properties study, and in each category the trend of progress of PEEK's composites is described based on the compound material.

### 4.1. *In Vitro* and* In Vivo* Bioactivity Study of PEEK Composite

Several studies have been conducted to probe the effect of compounding PEEK with bioactive materials on* in vitro* and* in vivo* bioactivity of the produced composite. PEEK/HA composites with different volume fraction of HA up to 40 vol% via injection molding method were evaluated* in vivo*. Preliminary histological* in vivo* study of composite with 20 vol% of HA showed the enhancement of the presence of fibroblast cells which stimulate vascularization. Osteoblastic activities study showed the formation of osteoid and osteocytes within lamellar bone in developing mature bone at longer implantation periods [15]. The SBF bioactivity test on HA/PEEK composites with different volume fraction up to 40% which were prepared by mixing of HA and PEEK powders, compaction, and sintering showed the higher rate of HA growth for the composite with higher volume fraction percentage of HA [14]. Biological study of HA/PEEK composites which were prepared by mixing and sintering the material powders using simple cubic mold shows the capability of this technique to replace the injection molding which is a high-cost method.* In vitro* study via SBF and cell seeding tests confirmed the bioactivity of the composite [79]. For better dispersion of HA particles in HA/PEEK composite nanosized HA (nHA)/PEEK with different nHA contents (15.1, 21.6, 29.2, and 38.2 vol%) was fabricated by Li et al. [80].* In vitro* study via SBF immersion, cell adhesion, and proliferation showed nanocomposite with 29.2 vol% of nHA content has the best bioactivity in comparison with other samples. For the improvement, the bonding between HA and PEEK of the HA/PEEK composite was fabricated via* in situ* synthetic method [81–83]. The biocompatibility study of* in situ* synthetic method for fabrication of composite showed the fabricated composites are nontoxic, and the bioactivity study showed the produced composites are bioactive.

Study of the bioactivity of *β*TCP-PEEK composite via injection molding method showed lower rates of osteoblast growth on the *β*TCP-PEEK compared to pure PEEK [84].* In vitro* study with osteoblast cells confirmed the nontoxicity of laser sintering method for producing *β*TCP/PEEK composite but showed no advantage of adding *β*TCP as fillers on cell growth [85, 86]. However,* in vivo* study of the laser sintered PEEK/*β*TCP implant revealed the PEEK/*β*TCP implants showed better interaction with surrounding bone and direct connection to the surrounding bone in comparison with pure PEEK [87].

*In vitro* study with osteoblast cells confirms the nontoxicity of laser sintering method for producing carbon black/PEEK composite but showed no advantage of adding carbon black as fillers on cell growth [85].* In vitro* study of HA/PEEK composite via selective laser sintering method showed improvement in bioactivity of the composite in comparison with pure PEEK. The results showed higher content of HA exhibited enhancement in cell proliferation and osteogenic differentiation [88].

*In vitro* osteoblast cell proliferation and viability study from PEEK, PEEK/carbon, PEEK/carbon/*β*TCP, and PEEK/carbon/bioglass 4s5S5 composites via laser sintering method revealed that all samples were nontoxic. However, the cell culture test did not show any advantageous effect of *β*TCP in the PEEK composite on the bioactivity properties of the samples. High-proliferation rates of osteoblasts on PEEK/carbon/bioglass composite showed the significant effect of bioglass on improving the bioactivity of the composite [86].* In vitro* study via MG-63 cells on glass fiber/PEEK composite showed a higher rate of cell proliferation on the surface of the composite compared to pure PEEK [89, 90].

Nano-TiO_2_ is another additive which is used for improvement in the bioactivity of PEEK composite.* In vitro* and* vivo* studies confirmed the positive effect of nano-TiO_2_ on improvement of bioactivity of PEEK.* In vitro* study demonstrated that compounding PEEK with nano-TiO_2_ was able to increase cell attachment and enhanced osteoblast cell spreading. In* in vivo* studies, the enhancement of the bone regeneration around the nano-TiO_2_/PEEK composite implant was observed by higher bone volume/tissue volume in comparison with the PEEK implant [20].

In another study of increasing the bioactivity of PEEK, strontium-containing hydroxyapatite/polyether ketone (Sr-HA/PEEK) composites were fabricated by compression molding technique.* In vitro* study involving apatite formation in SBF and MG-63-mediated mineralization confirmed higher bioactivity in comparison to HA/PEEK composite [16]. Also, calcium oxide and silicon dioxide (CS) were used as bioactive additives to PEEK composite.* In vitro* bioactivity study via SBF showed that by increasing the volume fraction of CS the bioactivity of the composite increased [91].  Table 4 summarizes the effect of the compound materials on the enhancement of the bioactivity of the PEEK composites.

### 4.2. Mechanical Properties of PEEK Composite

PEEK exhibits superb mechanical properties appropriate for load bearing orthopedic applications. However, as mentioned before the low mechanical properties of bioactive PEEK composites in comparison to PEEK are one of the biggest concerns of scientists and a lot of works in this field have been done. In this part, the present works based on the additives are described.

Studies showed that increasing the volume fraction of HA in the HA/PEEK composite increased Young's modulus and microhardness of the composite, though strength and strain at the fracture point decreased [76]. However, cyclic load on the PEEK/HA composite with different content of HA showed the HA/PEEK composite is a promising fatigue-resistant material for biomedical applications [92]. For improving the mechanical properties of the HA/PEEK composites the composites were prepared via* in situ* process. The composite showed strong physical bonding between HA and PEEK matrix due to improvement of mechanical properties of the composite in comparison with previously prepared HA/PEEK composites by other methods [81–83].

The mechanical properties of PEEK/HA nanoparticle composite showed the initial increase of tensile strength by increasing the content of HA nanoparticles to 5 vol% and after that decreasing the tensile strength. The authors described the first increase in tensile strength that was due to the “strong interactivity of nanoparticles and PEEK chains,” and they explained the agglomeration of HA nanoparticles for the contents of over 10 vol% which was due to decreased binding between nanoparticles and PEEK and reduction in the tensile strength of the composite [77, 78].

PEEK/HA whiskers composite via compression molding method showed the additive HA whiskers were oriented in the direction of viscous flow due to the production of composites with anisotropy mechanical properties. The results of mechanical properties showed an increase in the volume fraction of HA whisker reinforcement due to increased elastic modulus of the composite but caused a decrease in the ultimate tensile strength/strain at the failure point [18].

Polyether ketone (PEKK) reinforced with 0, 20, and 40 vol% HA whiskers specimens by compression molding method and subsequent annealing showed a decrease of the fatigue life with the increase in the volume fraction of the HA whiskers [93]. Effect of HA contents and mold temperature on the mechanical properties of PEKK/HA whiskers scaffolds was studied. The elastic modulus of the scaffold increased from 0 to 20 vol% HA with the increase of HA value from 20 to 40 vol%, while the yield strength and strain at the fracture point were decreased with increasing volume fraction of HA. Elastic modulus, yield strength, and yield strain were also increased by increasing the mold temperature [94].

The bending modulus of strontium-containing hydroxyapatite/polyether ketone (Sr-HA/PEEK) increased with increasing the volume fraction of Sr-HA. The elastic modulus of 25 vol% and 30 vol% Sr-HA reinforcement showed 113% and 136% increase, respectively, in comparison with pure PEEK. The bending strengths of 25 vol% and 30 vol% Sr-HA reinforcement showed 25% and 29% decrease, respectively, in comparison with pure PEEK [16].  Table 5 presents the summary of the effect of different compounds on the mechanical properties of the PEEK composite.

## 5. Summary and Conclusion

For long term load bearing implant applications, PEEK is the only commercial material that offers characteristics with good chemical resistance, radiolucency, and mechanical properties similar to those of human bones. However, bioactivity of PEEK is the biggest hindrance which causes reduction in the acceleration of worldwide spreading. We have summarized the previous study of bioactivation of PEEK and categorized them broadly to the bioactive PEEK composites and surface modified PEEK. The biggest concern about the PEEK composite is its mechanical properties. Thus, the PEEK bioactive composites were subcategorized to probe the previous studies from the bioactivity and mechanical aspects. Although different bioactive additives such as HA, Ti, TiO_2_, *β*-tricalcium phosphate, and bioactive glass improve the bioactivity of PEEK's composite, the low mechanical properties of PEEK's composite are still its most important weakness. The surface modification of PEEK for biomedical application was subcategorized based on the techniques which were used for modifying the surface of the PEEK's implants. Between these methods the deposition of HA via plasma spraying method is the only method which qualified for commercial usage. However, there are still some concerns with this method such as damaging the surface chemistry of PEEK substrate and therefore in-depth research is needed. The trend of research in the bioactivity of PEEK shows a very encouraging result which has potential to overcome the existing problems in the current techniques and production of bioactive PEEK implant and spreading its application as bioactive material in orthopedic and dental implant areas.

## Figures and Tables

**Table 1:** Deposited functional groups on PEEK via wet chemical deposition.

Functional group	Results
–ONa	Enhancement of wettability [31]. Enhancement of apatite formation [34].

–OH	Enhancement of wettability [31]. Disable to graft to Fibronectin [32].

–F	Enhancement of wettability [31].

–OH(CFCl_3_)	Enhancement of wettability [31].

Amine	Improvement of cellular adhesion and growth [28].

Carboxyl	Improvement of cellular adhesion and growth [28].

–NH_2_	Disable to graft to Fibronectin [32].

–NCO	Fibronectin covalently grafted to PEEK-NCO [32].

Fibronectin grafting	Enhancement of adhesion and spreading of Caco-2 cells in the absence of serum in comparison with PEEK substrates, which were simply coated with FN [33].

–SO_3_H	Producing 3D nanostructured treated layer. *In vitro* (cell culture and apatite formation) and *in vivo* study showed enhancement of bioactivity [22].

**Table 2:** Different ionization assistants which have been used for improving the bioactivity of PEEK via plasma treatment.

Ionization assistant	Results
Oxygen	Enhancement of wettability [35, 36]. Increase of push-out force and bone-implant contact area [41].

Air	Enhancement of wettability [35, 36].

Nitrogen	Enhancement of wettability [35, 36].

Ammonia	Enhancement of wettability [35, 36].

Argon	Enhancement of wettability [35, 36]. Using vapor as a plasma resource showed improvement of osteoblast adhesion, spreading, proliferation, and early osteogenic differentiation [42]. Increasing surface roughness, enhancement of cell adhesion, proliferation, and metabolic activity [43].

N_2_/O_2_	*In vitro* study via osteoblast cells showed no disadvantages on cell viability [37].

NH_3_	Enhancement of wettability and increasing cell growth [38].

Ammonia/argon	Enhancement of cell proliferation rate and enhancement of wettability [39].

Hydrogen/argon	Enhancement of cell proliferation rate and enhancement of wettability [39].

CH_4_/O_2_	Enhancement of cell adhesion and enhancement of wettability [40].

O_2_/Ar	Enhancement of cell adhesion, proliferation, and osteogenic differentiation of adMSC [40].

NH_4_	Enhancement of cell adhesion, proliferation, and osteogenic differentiation of adMSC [40].

**Table 3:** Summary of the existing deposition methods/materials for improving PEEK bioactivity.

Deposited material	Deposition method	Area of studies	Findings
HA	Plasma spray	—	Low adhesion of the coating layer to the substrate [54].
	Vacuum-plasma-sprayed	Using titanium intermediate coating layer	Good interlocking between PEEK substrate and intermediate Ti layer and preventing damage of the substrate [55].
	Radio frequency magnetron sputtering	Crystalline YSZ layer was deposited as an intermediate layer	Enhancement crystallinity of HA deposited layer during sintering [56].
	Plasma spraying	Crystallographic compositions, adhesions, and microstructures of HA coating via plasma spraying method on different PEEK (unfilled and CF/PEEK) specimens were studied and compared with HA coating on Ti-6Al-4V	Almost the same structure of HA coatings for PEEK and Ti-6Al-4V substrate. Plasma spraying method does not have a negative effect on mechanical properties of PEEK [57].
	Vacuum-plasma-sprayed	*In vitro* study with human bone marrow mesenchymal stem cells and *in vivo* study	Viability improvement and enhancement of cell differentiation and proliferation. Promoting of bone growth [58].
	Aerosol deposition	Microstructure, *in vivo*, *in vitro* study	Dense microstructure with no pores and cracks. Enhancement of bioactivity in terms of cell proliferation, differentiation, adhesion morphology, and bone-implant contact ratio [59].
	Spin coating	*In vivo* osseointegration (histomorphometry) study	Improvement of bone-to-implant contact area [60].
	Chemical deposition	–SO_3_H functional group was created via sulphonation and HA crystalline particles were chemically deposited	The proposed method did not use high temperature and improved the wettability [61].

A-TiO_2_ and R-TiO_2_	Arc ion plating	*In vitro* SBF immersion and osteocompatibility study	Enhancement of apatite formation and improvement of osteocompatibility, in which R-TiO_2_ achieves the best result [63].

TiO_2_	Arc ion plating	*In vitro* osteoblast study	Improvement in cell adhesion, proliferation, and differentiation [21].

TiO_2_/BMP-2	Immobilization	*In vivo* study	Enhancement of bone-to-implant contact ratio in comparison with TiO_2_ and BMP-2 coating layer and bare PEEK [64].

Ti	Plasma spray	*In vivo* study	Enhancement bone-to-implant contact ratio [65].
	Electron beam deposition	*In vitro* study in terms of proliferation and differentiation of MC3T3-E1 cells and *in vivo* study	Enhancement of *in vitro* bioactivity and bone-to-implant contact ratio [6].
	VPS	Probing the effect of pretreatment of the substrate with NaOH solution on bioactivity via *in vitro* SBF immersion study	Improvement bioactivity in terms of apatite formation [66].
	PVD and VPS	*In vivo* comparative study for probing the effect of PVD and VPS methods on the Ti deposited on CF/PEEK substrate	No significant difference between these two methods in terms of bioactivity [67].
	PVD	*In vivo* study of Ti-coated CF/PEEK for dental implant application	Coated samples showed better bone growth around the coated implant but the same push-out force for coated and uncoated samples by new bone growth [68].
	Electron beam deposition	Wettability, *in vitro* study via MC3T3-E1 cell and *in vivo* study	Enhancement of *in vitro* bioactivity and bone-in-contact ratio [6].

Zirconium and titanium tetra	PVD	*In vitro* study via osteoblast	Enhancement of osteoblast cell growth [69].

DLC	Plasma immersion ion implantation and deposition	*In vitro* study via osteoblast	Enhancement of attachment, proliferation, and differentiation of osteoblast [70].

**Table 4:** Effect of the compound materials on the bioactivity of the PEEK composite.

Compound material	Studied areas	Results
HA	Probing the effect of HA volume fraction on bioactivity via *in vivo* study.	Enhancement of the presence of fibroblast cells, formation of osteoid and osteocytes within lamellar bone [15].
	Probing the effect of HA volume fraction on bioactivity via SBF immersion test.	Higher rate of HA growth for the composite with higher volume fraction of HA [14].
	*In vitro* study of the new method of simple cubic molding and sintering.	Confirmed improvement of bioactivity of the composite [79].
	Biocompatibility and bioactivity study of the produced composite via *in situ* synthetic method.	Produced composite showed nontoxic and the bioactive properties [81–83].
	*In vitro* bioactivity study of HA/PEEK composite produced by selective laser sintering method.	Improvement in bioactivity of the composite and higher content of HA exhibited higher bioactivity rate [88].

nHA	Probing the effect of nHA volume fraction on bioactivity via *in vitro* study by SBF immersion, cell adhesion, and proliferation.	Nanocomposite with 29.2 vol% of nHA content showed the best bioactivity in comparison with other samples [80].

*β*TCP	*In vitro* bioactivity study via osteoblast cells.	Lower rates of osteoblast growth on the *β*TCP-PEEK compared to pure PEEK [84].
	Biocompatibility study of laser sintering method for producing *β*TCP/PEEK via *in vitro* study by osteoblast cells.	Confirmed nontoxicity of laser sintering method for producing *β*TCP/PEEK composite but showed no advantage of adding *β*TCP as an additive on cell growth [85, 86].
	*In vivo* bioactivity study of the laser sintered PEEK/*β*TCP composite.	Better interaction with surrounding bone and direct connection to the surrounding bone [87].

Carbon black	Biocompatibility study of laser sintering method for producing carbon black/PEEK composite via* in vitro* study by osteoblast cells.	Confirmed nontoxicity of laser sintering method for producing carbon black/PEEK composite but showed no advantage of adding carbon black as an additive on cell growth [85].

Carbon, carbon/*β*TCP, and carbon/bioglass 4s5S5	Biocompatibility and bioactivity study of produced composites via laser sintering method.	Produced composite via laser sintering method was nontoxic. PEEK/carbon/bioglass composite showed improvement in the bioactivity property [86].

Glass fiber	*In vitro* study via MG-63 cells.	Higher rate of cell proliferation [89, 90].

Nano-TiO_2_	*In vitro* and *in vivo *study.	Increasing in cell attachment and enhanced osteoblast cell spreading. Enhancement of the bone regeneration around the nano-TiO_2_/PEEK composite [20].

Sr-HA	*In vitro* study contains apatite formation in SBF and MG-63-mediated mineralization.	Enhancement of bioactivity [16].

CS	Probing the effect of CS volume fraction on bioactivity via *in vitro* bioactivity study by SBF immersion.	By increasing the volume fraction of CS the bioactivity of the composite increased [91].

**Table 5:** Effect of the compound materials on the mechanical properties of the PEEK composites.

Compound material	Studied mechanical properties	Results
HA	E, microhardness, ultimate tensile strength/strain	Young's modulus and microhardness of composite increased, ultimate tensile strength and strain at the fracture point decreased [76].
	Fatigue-resistant	Showing enough fatigue-resistant property for biomedical applications [92].
	Ultimate tensile strength	Prepared composite via *in situ* process showed strong physical bonding between HA and PEEK matrix and enhanced ultimate tensile strength [81–83].

HAnp	Ultimate tensile strength	Initial increase of tensile strength by increasing HAnp content to 5 vol% and after that decreasing the tensile strength [77, 78].

Whiskers HA	E, isotropy property, ultimate tensile strength/strain	Anisotropy mechanical properties, increasing of E and decreasing in the ultimate tensile strength/strain by increasing of the volume fraction of HA whisker reinforcement [18].
	Fatigue life	Decreasing of the fatigue life with increase in the volume fraction of the HA whiskers in PEKK [93].
	E, ultimate strength and strain	Elastic modulus increased, while the ultimate tensile strength and strain decreased with increasing volume fraction of HA. Elastic modulus, yield strength, and yield strain were increased by increasing the mold temperature [94].

Sr-HA	E, bending strength	The bending modulus, elastic modulus increased with the volume fraction ratio of Sr-HA. The elastic modulus of 25 vol% and 30 vol% Sr-HA reinforcement showed 113% and 136% increase, respectively, in comparison with pure PEEK. The bending strengths of 25 vol% and 30 vol% Sr-HA reinforcement showed 25% and 29% decrease, respectively, in comparison with pure PEEK [16].
